# Thalamo-cortical network activity between migraine attacks: Insights from MRI-based microstructural and functional resting-state network correlation analysis

**DOI:** 10.1186/s10194-016-0693-y

**Published:** 2016-10-24

**Authors:** Gianluca Coppola, Antonio Di Renzo, Emanuele Tinelli, Chiara Lepre, Cherubino Di Lorenzo, Giorgio Di Lorenzo, Marco Scapeccia, Vincenzo Parisi, Mariano Serrao, Claudio Colonnese, Jean Schoenen, Francesco Pierelli

**Affiliations:** 1 Research Unit of Neurophysiology of Vision and Neurophthalmology, G.B. Bietti Foundation-IRCCS, Via Livenza 3, 00198 Rome, Italy; 2 Department of Neurology and Psychiatry, Neuroradiology Section, “Sapienza” University of Rome, Rome, Italy; 3 Department of Medico-Surgical Sciences and Biotechnologies, Neurology Section, “Sapienza” University of Rome, Rome, Italy; 4 Don Carlo Gnocchi Onlus Foundation, Milan, Italy; 5 Laboratory of Psychophysiology, Psychiatric Clinic, Department of Systems Medicine, University of Rome “Tor Vergata”, Rome, Italy; 6 Department of Medico-Surgical Sciences and Biotechnologies, “Sapienza” University of Rome Polo Pontino, Latina, Italy; 7 IRCCS Neuromed, Pozzilli, (IS) Italy; 8 Headache Research Unit, Department of Neurology-CHR Citadelle, University of Liège, Liège, Belgium

**Keywords:** Migraine, Thalamus, Resting state, Fractional anisotropy, Magnetic resonance imaging

## Abstract

**Background:**

Resting state magnetic resonance imaging allows studying functionally interconnected brain networks. Here we were aimed to verify functional connectivity between brain networks at rest and its relationship with thalamic microstructure in migraine without aura (MO) patients between attacks.

**Methods:**

Eighteen patients with untreated MO underwent 3 T MRI scans and were compared to a group of 19 healthy volunteers (HV). We used MRI to collect resting state data among two selected resting state networks, identified using group independent component (IC) analysis. Fractional anisotropy (FA) and mean diffusivity (MD) values of bilateral thalami were retrieved from a previous diffusion tensor imaging study on the same subjects and correlated with resting state ICs Z-scores.

**Results:**

In comparison to HV, in MO we found significant reduced functional connectivity between the default mode network and the visuo-spatial system. Both HV and migraine patients selected ICs Z-scores correlated negatively with FA values of the thalamus bilaterally.

**Conclusions:**

The present results are the first evidence supporting the hypothesis that an abnormal resting within networks connectivity associated with significant differences in baseline thalamic microstructure could contribute to interictal migraine pathophysiology.

## Background

During recent years, various experimental data suggested that the functional state of the migraineur’s brain is altered between attacks. This was initially observed with clinical neurophysiology methods that disclosed for instance interictal deficient habituation of sensory responses attributable to abnormal thalamo-cortical interactions [1, 2], and abnormal brain responses to various neuromodulatory techniques [3]. Interictal functional abnormalities were confirmed also with functional imaging methods in response to both noxious [4, 5] and innocuous stressors [6, 7]. Recently, several independent research groups showed that changes can also be demonstrated at rest, i.e. without any sensory input, in the microstructure of several brain areas [8], including the thalamus [9, 10]. The conjunction of neuroimaging and neurophysiological data can be considered as robust evidence favouring morphological and functional brain alterations as prominent features of migraine pathophysiology. Since the brain areas are part of interconnected cortical and subcortical networks, it seems of major interest to analyse during the interictal phase the functional connectivity between brain networks at rest and its relationship with the thalamic microstructure.

Among the various neuroimaging procedures, resting state functional MRI (RS-fMRI) analyses the spontaneous BOLD signal modulations not attributable to explicit inputs or outputs [11] and allows studying among which distributed brain areas activity at rest is related [12]. A common method to identify spatial patterns of coherent spontaneous BOLD activity, so-called functional connectivity, is independent component analysis (ICA). ICA is a high-order statistical method to examine functional connectivity by deconvolving the cerebral signals into components that are maximally independent and that reflect specific interconnected neuroanatomical networks. Compared to other methods, ICA is devoid of any *a-priori* definition of seed regions of interest [11]. RS-fMRI ICA allows the study of group differences in the temporal relationship among independent spatially distributed networks/components [12]. With this method, spontaneous brain activity was shown to be organized in specific and distinct spatial patterns, or sets of resting-state networks [13, 14].

In recent years, several fMRI studies have assessed resting state functional connectivity in various networks in migraine patients. Most of them have used an *a-priori* selected seed-based analysis [15–21]. Between selected brain areas of the default mode network (DMN) both increased [20, 22] or decreased [23] connectivity was reported. Two studies by the same group of researchers used the single independent component approach without *a-priori* hypothesis [24, 25]. They found evidence for reduced DMN [25] and executive control network [24] connectivity in migraine without aura patients between attacks. To the best of our knowledge, there are no RS-fMRI studies using ICA to determine the functional connectivity between networks (not within) between migraine attacks. Moreover, RS-fMRI studies of subcortical and cortical nodes were not combined up to now with DTI studies to analyse in migraine patients the connectivity patterns between the thalamus and various functional cerebral networks at rest. We decided, therefore, to use ICA of the whole brain to search for changes in functional connectivity maps at rest in interictal episodic migraine without aura patients. In addition, the thalamo-cortical network was statistically inferred by correlating selected resting state independent component activity strength and thalamic anisotropy.

## Methods

### Subjects

We initially enrolled 32 episodic migraine patients without aura (MO, ICHD-3beta code 1.1) who attended our headache clinic in a time period of 2 years and agreed to undergo MRI. We discarded recordings of 14 patients who had an attack within 3 days before or after the recording session.

The final analysis dataset comprises thus 18 right-handed MO patients [26] who subsequently participated in a comprehensive battery of neuroimaging tests, including RS-fMRI. We have published elsewhere the results of the diffusion tensor imaging and voxel based morphometry studies performed on the initial 14 patients and used these data combined with those of 6 additional patients to search for correlations with RS-fMRI data [9, 27]. Patients underwent MRI scans during the interictal period (MO), defined as an absence of migraine attacks for at least three days before and after MRI. Inclusion criteria were as follows: no history of other neurological diseases, systemic hypertension, diabetes or other metabolic disorders, connective or autoimmune diseases, and any other type of primary (including chronic migraine) or secondary headache. Patients had uni/bilateral migraine headaches, but not fixed pain on the same side. In order to avoid confounding effects on neuroplasticity due to pharmacologic treatment, no preventive anti-migraine drugs were allowed during the preceding 3 months. The control group comprised 19 right-handed healthy volunteers (HV) made up of medical school students and healthcare professionals of comparable age and gender distribution to the experimental group. Control subjects did not have any overt medical conditions, personal or family history of migraine or epilepsy, or take regular medication. Female subjects were always scanned at mid-cycle. All scanning sessions were performed in the afternoon (4.00–7.00 p.m.).

None of the enrolled subjects had sleep deprivation or alcohol consumption the day preceding the scans. Caffeinated beverages were not allowed on the day of scanning. Further exclusion criteria for both HV and MO were evidence of brain lesions on structural magnetic resonance imaging. All participants received a complete description of the study and granted written informed consent. The ethical review board of the Faculty of Medicine, University of Rome, Italy, approved the project.

### Imaging protocols

All images were acquired using a Siemens 3 T Verio MRI scanner with a 12-channel head coil and structural anatomic scans were performed using T1-weighted sagittal magnetization-prepared rapid gradient echo (MP-RAGE) series (TR: 1900 ms, TE: 2.93 ms, 176 sagittal slices, 0.508 x 0.508 x 1 mm^3^ voxels).

We acquired an interleaved double-echo Turbo Spin Echo sequence proton density and T2-weighted images (repetition time: 3320 ms, echo time: 10/103 ms, matrix: 384 × 384, field of view: 220 mm, slice thickness: 4 mm, gap: 1.2 mm, 50 axial slices).

Functional MRI data were obtained using a T2*-weighted, echo-planar imaging (TR: 3000 ms, TE: 30 ms, 40 axial slices, 3.906 x 3.906 x 3 mm, 150 volumes).

Functional BOLD data were collected in a 7 min 30 s run, during which subjects were instructed to relax, avoid motion and keep their eyes closed.

### Data processing and analyses

Image data processing was performed on a personal computer using statistical parametric mapping (SPM8) software package (Wellcome Trust Centre for Neuroimaging, London, UK; http://www.fil.ion.ucl.ac.uk/spm), GIFT v3.0 and FNC toolbox (http://mialab.mrn.org/) in Mat-Lab (http://www.mathworks.com/). The overall image data processing is based on the method already described elsewhere [12].

All images from a single subject were realigned using a 6-parameter rigid body process, replaced by a cubic spline interpolation. We did not perform slice time correction. Slice-timing correction is not mandatory because the haemodynamic response is longer than TR (about 30 s). Moreover, the actual EPI uses multiband sequences with simultaneous echo refocusing and parallel imaging (called GRAPPA by Siemens), that makes slice time correction obsolete. The structural (T1 – MPRAGE) and functional data were coregistered for each participant dataset, normalized into the standard Montreal Neurological Institute space, and then transformed into a common stereotactic space based on Talairach and Tournoux [28]. Finally, functional images were spatially smoothed with an 8 mm full width half-maximum Gaussian kernel on each direction.

### Component identification and selection

Grouped spatial ICA was performed for all 37 participants (HV + MO) using the infomax algorithm [29]. Two separate group spatial ICAs were also carried out in HV and MO patients to ensure that the fluctuations of components at rest in each group of subjects were similar to those obtained in the total group of 37 subjects. GIFT software automatically decomposed data into 39 components. A modified version of the minimum description length (MDL) criterion was adopted to determine the number of components from the aggregate data set [30]. Single subject spatially or temporally independent maps were then back-reconstructed from the aggregate mixing matrix [31].

All 39 components were inspected after plotting to templates in GIFT, using *a priori* probabilistic maps, and those of interest whose patterns mainly consisted of gray matter rather than non-gray matter were selected. Components located in CSF or white matter, or with low correlation to gray matter, can be artifacts, such as eye movements, head motion, ballistic artifacts, and were discarded. With FNC toolbox in MatLab, after removing all the artifactual components and applying a *p*-value threshold of 0.01 (false discovery rate corrected), only two components survived for further analysis. Before performing correlation analyses, a band-pass Butterworth filter between 0.033 Hz and 0.13 Hz was applied on the two selected component time courses. Each IC consists of a temporal waveform and an associated spatial map; the latter is expressed in terms of Z-scores that reflect the degree to which a given voxel time-course correlates with the specific IC temporal waveform, i.e. a way to quantify the strength of the IC [32]. As a further step and in order to search for a correlation between regional RS-fMRI network changes and clinical features, the Z-max scores (voxel-wise analysis) of each IC network were extracted for each participant.

### Diffusion weighted imaging of the thalami

**Fig. 1:**
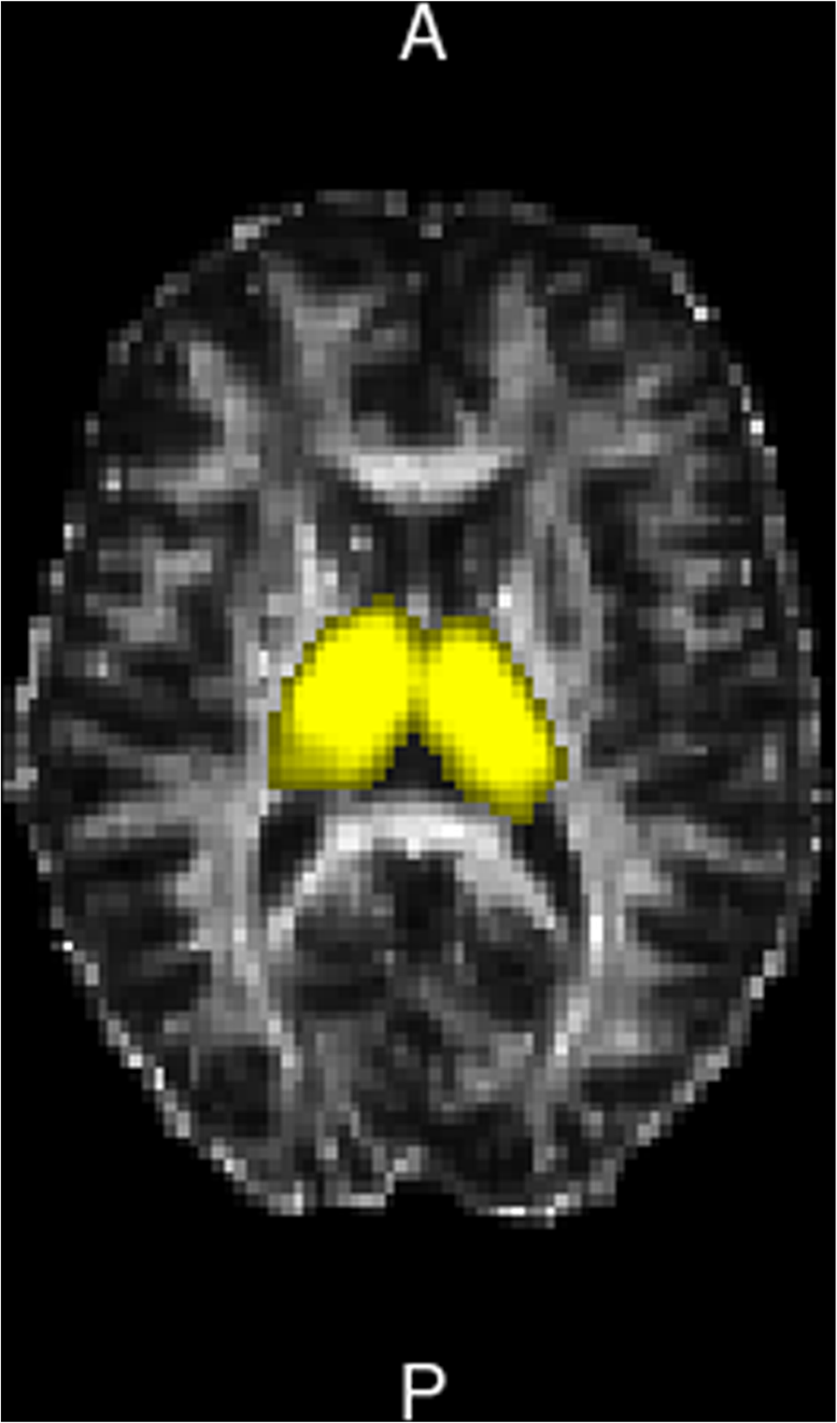
Exemplary single subject axial representation of the diffusion tensor imaging FA map with the analyzed thalamic ROIs highlighted in yellow

Diffusion tensor imaging (DTI) was acquired using single shot echo-planar imaging, with a 12–channel head coil (TR 12200 ms, TE 94 ms, 72 axial slices, 2 mm thickness, isotropic voxels). Images from the same participants and during the same session were obtained with diffusion gradients applied along 30 non-collinear directions, effective b values of 0 and 1000 s/mm2 were used. Image data processing was performed with the FSL 4.0 software package (FMRIB Image Analysis Group, Oxford, England). Diffusion data were corrected for motion and distortions caused by eddy current artifacts; FMRIB's Diffusion Toolbox (FDT) was used for local fitting of diffusion tensors, and fractional anisotropy (FA) and mean diffusivity (MD) maps were created. Two regions of interest (ROI, from the “Harvard-Oxford Subcortical Structural Atlas” as distributed with FSL) were defined for each subject, covering totally right and left thalami on each slice (Fig. 1). The medial boundaries were determined on each slice using cerebro spinal fluid as a landmark; lateral limits were verified using FA maps to exclude the internal capsule. Mean FA and MD in each region for every subject were obtained by averaging the values of those voxels contained in the ROI.

We have previously published *in extenso* the results of the DTI analyses performed on the first 29 subjects, 15 HV and 14 MO [9].

### Statistical analyses

Group differences for demographic data were estimated using ANOVA test.

We used a 2-sample t-test to detect significant differences in correlation values between the two independent components for HV vs MO. A conservative *p* value of *p* < 0.05 (correction for multiple comparisons with false discovery rate selected) was used as significance cut-off. Connectivity combinations with statistically significant (*p* < 0.01) lag values were assessed using a two sample t-test of the difference between averaged HV and MO patient lags. As a further step and in order to search for a correlation between regional RS-fMRI network changes and clinical features, the Z-max scores of each IC network were extracted for each participant.

Finally, we used Pearson’s test to search for correlations between the MR DTI parameters FA and MD, individual IC Z-max scores and clinical variables such as severity of headache attacks [0–10], duration of migraine history [years], monthly attack frequency [n], attack duration [hours], days elapsed since the last migraine attack [n]. *P* values ≤ 0.05 were considered to indicate statistical significance.

## Results

All subjects completed the study. Demographic and clinical data for the two groups are summarized in Table 1. Structural brain MRIs were normal in all participants. Specifically in migraine patients there were neither white matter lesions nor features suggestive of cortical atrophy.

**Table 1:** Clinical and demographic characteristics of healthy volunteers (HV) and migraine patients without aura scanned between (MO) attacks. Data are expressed as means ± SD

	HV	MO
	(*n* = 19)	(*n* = 18)
Women (n)	12	12
Age (years)	29.7 ± 4.0	32.4 ± 7.2
Duration of migraine history (years)		14.1 ± 6.8
Attack frequency/month (n)		3.1 ± 2.1
Attack duration (hours)		27.3 ± 30.0
Visual analogue scale (n)		7.3 ± 0.9
Days from the last migraine attack (n)		20.8 ± 18.5

### Resting state fMRI

**Fig. 2:**
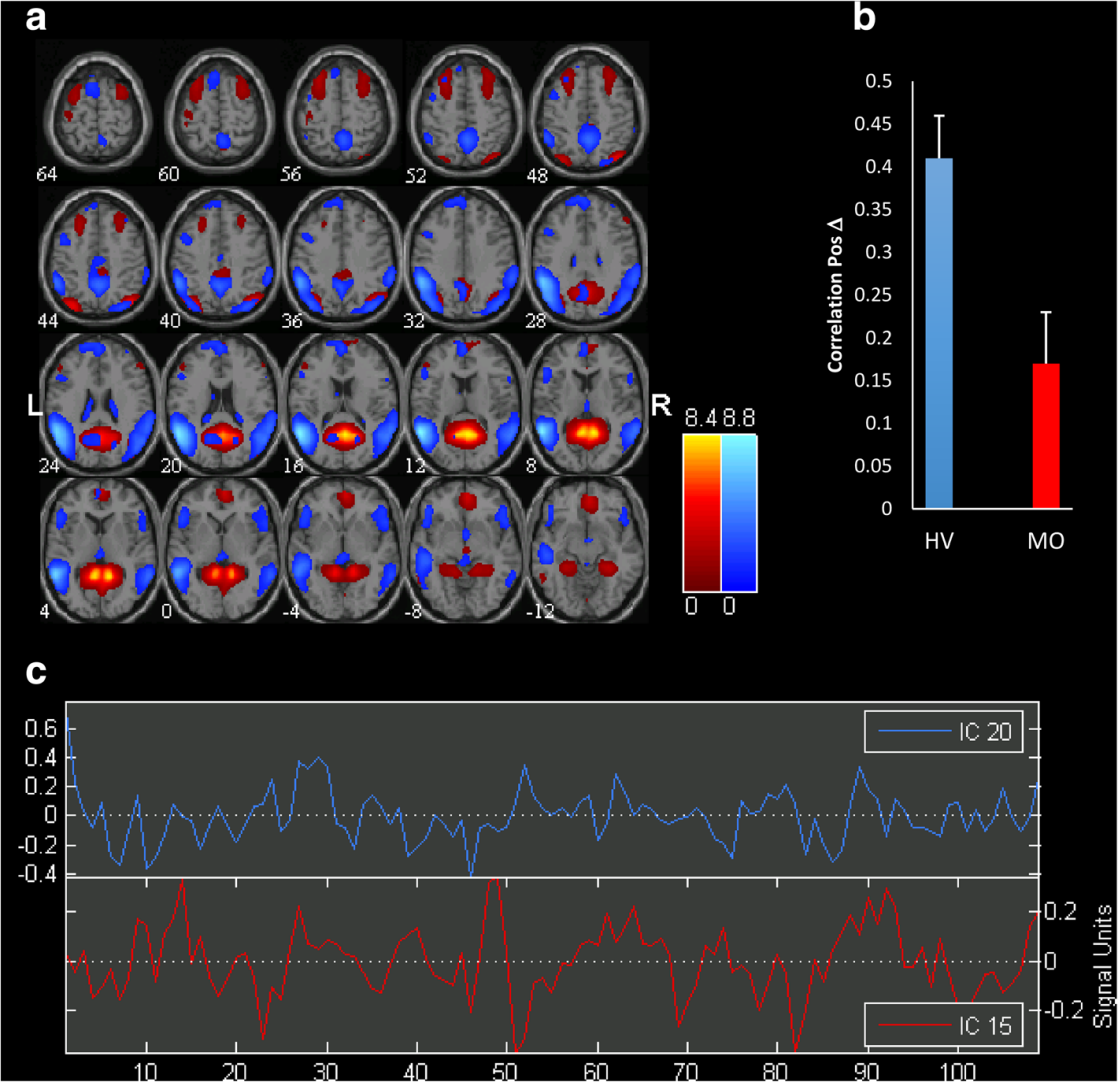
**a** Representation of the two significant Independent Components (IC) functional connectivity networks differing in migraine patients scanned between attacks (MO) compared with healthy volunteers (HV) separated by independent component analysis (ICA). All images have been coregistered into the space of the MNI template. Brain areas are respectively coloured in hot metal scale (IC15) or in azure-blue (IC20). The numbers beneath each image refer to the z coordinate in Talairach space. **b** The bar graph reflects FDR corrected correlation between the 2 ICs, *p* < 0.05, in HV and MO. **c** Time course of spontaneous blood oxygen level dependent (BOLD) activity recorded during resting state and extracted from each of the two significant ICs

Significantly correlated components are represented in Fig. 2.

We found a significant difference in functional connectivity between independent components IC20 and IC15 in migraine patients between attacks respective to healthy volunteers. In fact, the correlation of independent component pair (IC20-IC15) is significantly lower in MO compared to HV (rho = 0.17 in MO vs rho = 0.41 in HV; FDR corrected, *p* < 0.05).

These components encompass respectively interconnected areas of the so-called default mode network (IC20) and a network composed of the visuo-spatial system and medial visual cortical areas (IC15), as seen on GIFT templates.

Although component directions slightly differed between HV and MO patient group, lag difference did not reach the level of significance.

There was no significant correlation between ICs Zmax-scores and clinical data.

### Diffusion Tensor Imaging (DTI) data

The DTI results confirmed those published elsewhere on the first 29 subjects [9]. In MO patients the bilateral thalami have shown a significantly increased fractional anisotropy (F_(1,35)_ = 4.99, *p* = 0.03 and F_(1,35)_ = 4.86, *p* = 0.03 for the left and right thalamus respectively), but only a tendency to mean diffusivity change (F_(1,35)_ = 2.50, *p* = 0.12 and F_(1,35)_ = 3.44, *p* = 0.07 for the left and right thalamus respectively), than in HV.

### Thalamo-cortical network correlation analysis

**Fig. 3:**
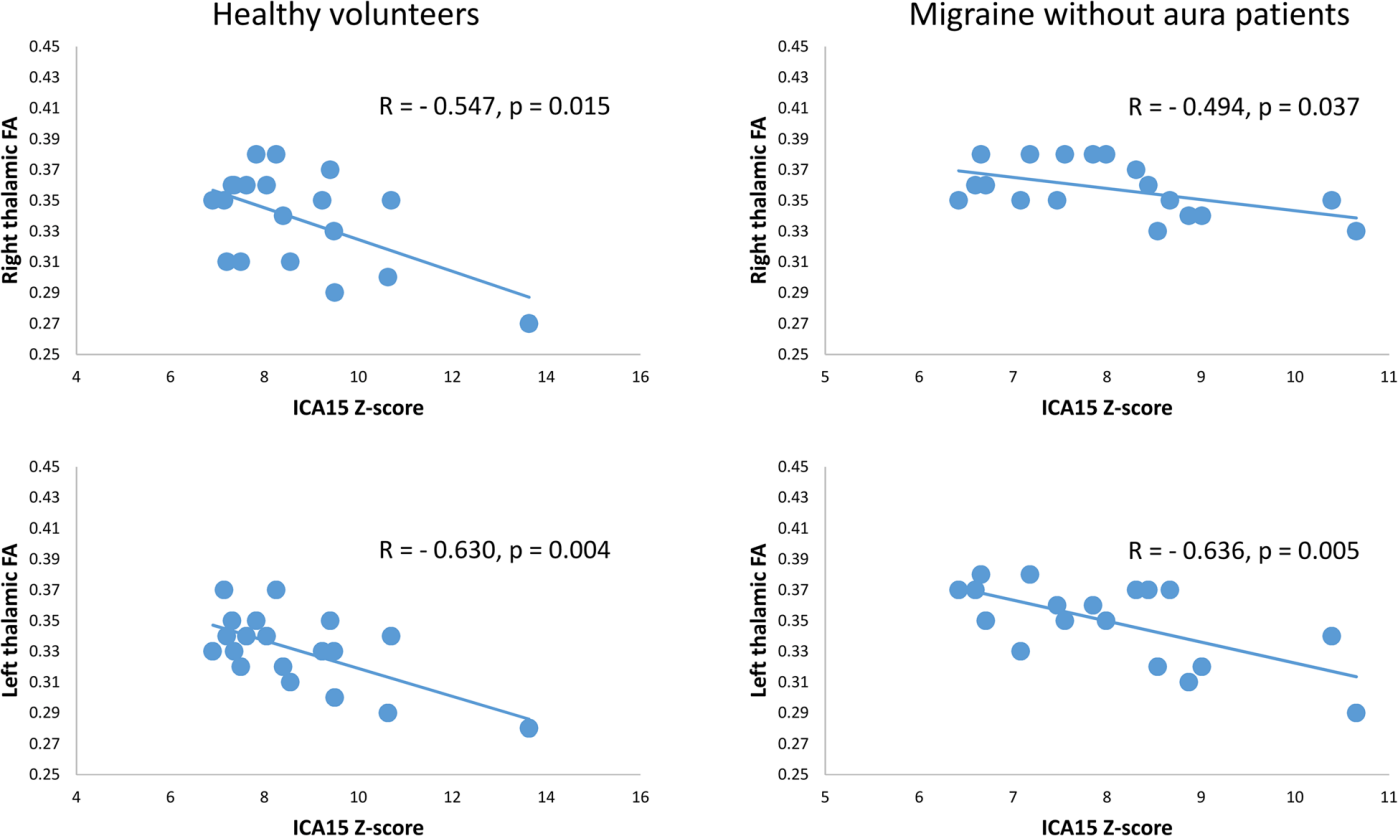
In both HV (left panel) and MO (right panel) groups, Z-score of the independent component (IC)15, encompassing the visuo-spatial system and medial visual cortical areas, correlated negatively with bilateral thalamic fractional anisotropy (FA) values

Pearson’s correlation test disclosed that the IC15 Z-score correlated negatively with bilateral thalamic FA values in both HV (right *r* = − 0.547, *p* = 0.015; left r = − 0.630, *p* = 0.004) and MO (right *r* = − 0.494, *p* = 0.037; left *r* = − 0.636, *p* = 0.005) (Fig. 3).

## Discussion

Our study was specifically designed to search for differences in resting state networks and to test whether the resulting networks are correlated with thalamic microstructure in interictal migraine state. We found that functional connectivity of networks involved in in information processing, in cognitive, emotional, and, especially visual, attention processes differ between patients and healthy volunteers. We will discuss the possible neurobiological underpinnings of our findings and their potential relevance for migraine pathophysiology.

### Resting state functional connectivity between attacks

Functional connectivity between the default mode network (IC 20) and a network composed of the visuo-spatial system and medial visual cortical areas (IC 15) is significantly reduced in migraine patients between attacks compared to HV.

The so-called default-mode network (DMN) encompasses a set of regions with relatively greater activity during “rest” than during active conditions [33]. The DMN includes the posterior cingulate gyrus, precuneus, medial prefrontal cortex, angular gyrus, and medial temporal lobe regions including the hippocampus and the lateral temporo-parietal area [34]. Although the exact functional role of the DMN is not completely understood, it is thought to be involved in retrieval of information from long-term memory and its manipulation for optimizing the sensorium and for problem solving and planning. The DMN is thus important in conscious experience as well as in maintaining a general low-level focus of attention for an event [35, 36]. Although the DMN is anatomically and functionally distinct from networks involved in sensory functions, comparative studies in animal and human studies have shown that it is functionally correlated with all other resting state networks [14]. The visuo-spatial system comprises the posterior parietal cortex at the occipito-parietal junction, the precuneus, the posterior cingulate cortex and the frontal pole [13, 37]. Activity within this network in the resting state is associated with gathering information about our outer, and possibly inner, world [38], in episodic memory recall and orienting attention to salient novel or familiar stimuli and in emotional processing associated with episodic memory [39]. The medial visual cortical areas include the primary visual cortex as well as medial extrastriate regions such as lingual gyrus, inferior division of precuneus and lateral geniculate nucleus [13, 14]. This network is supposed to play a role in episodic memory, visual and visuo-spatial processing, reflections upon self and aspects of consciousness.

Given the brain networks involved and their known functions, the abnormalities we found in interictal migraine suggest a dysfunction of information gathering, evaluation and integration, and impaired short- as well as long- term memory processes. Moreover, because of the predominant involvement of visual areas/systems visuo-perceptual and visuo-spatial integration could be impaired in migraine between attacks. Whether these resting state fMRI abnormalities are the connectivity correlates of the subtle impairments in neuropsychological performances, such as processing speed, verbal memory, and physiognomy recognition, previously reported in migraine between attacks remains to be determined [40–42]. It was shown that resting state spontaneous brain activity can be used to predict the task-response properties of brain regions [43]. It is thus of interest to verify if the reduced spontaneous network activity found here is correlated with the abnormal cognitive evoked potentials reported in migraine patients [44–49].

### Thalamo-cortical interactions

We previously argued [9] that, in MO patients scanned between attacks, the pattern of increased anisotropy associated with normal MD may reflect shrinking of neuronal and glial cells and/or gain of directional organization in combination with a preserved cell density [50, 51]. Interestingly, data from animal models showed that cell shrinking may coincide with a reduced neuronal electric response [52, 53]. Therefore, since grey matter in the single thalamic sub-nuclei does not have a unique oriented fibre structure, the increased FA found in between attacks might also result from a decrease in neuron connections and thus dendritic arborization, which in turn may result in a reduced number of local circuits [54].

Here, the most striking finding is the correlation between MRI diffusion-weighted features of the thalamus and the functional connectivity between brain networks. In both HV and migraineurs between attacks, individual Z-scores of IC15, containing the visuo-spatial system and medial visual areas, correlated negatively with FA values of bilateral thalami, suggesting that the lower is the between networks connectivity the higher is the FA in the thalami bilaterally. However, this association between thalamic diffusion parameters and RS-fMRI is evident in examining each individual group, suggesting that the correlation between thalamic microstructure and RS-fMRI is a more general phenomenon related to the connectivity mechanisms. Nevertheless, we found significant differences in baseline thalamic microstructure – increased FA in MO – and in within networks connectivity – decreased connection in MO – between patients and healthy controls.

**Fig. 4:**
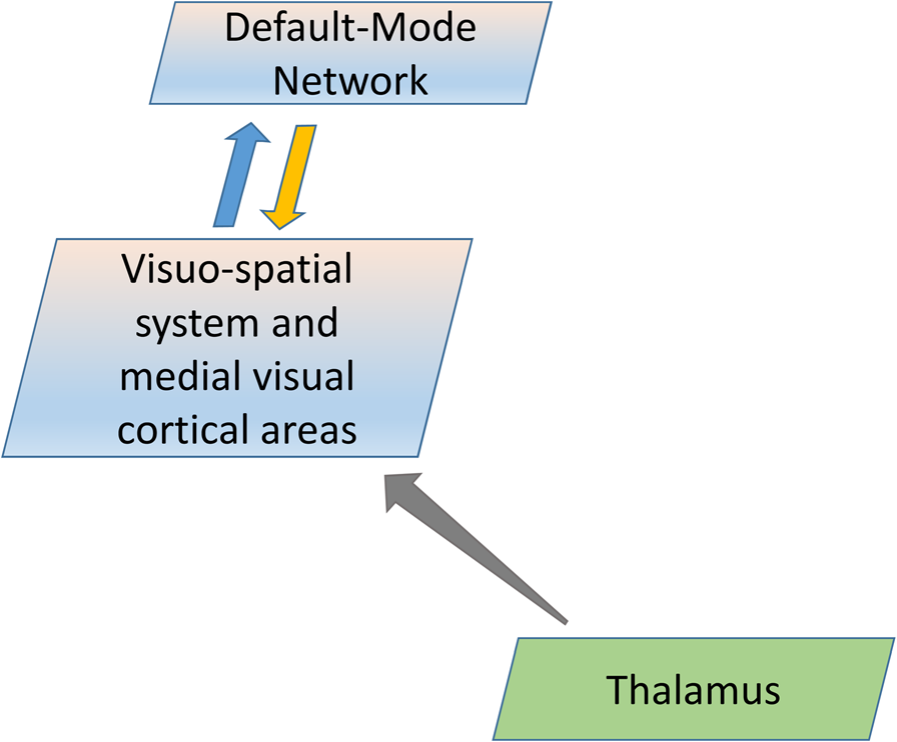
Schematic representation of information flow describing the thalamo-cortical neural network model that can encompass the present findings in migraine patients scanned between attacks. During the interictal period, a reduced thalamic activity, as highlighted by an increased FA, activates less the visuo-spatial system and medial visual cortical areas, which in turn deactivate the DMN network

Overall, the results from the correlation analysis fit strikingly with evidence coming from neuroimaging studies showing a distinct functional connectivity between the thalamus and several areas within the visuo-spatial system and medial visual areas (e.g. posterior cingulate cortex, visual cortex, precuneus) [55–57]. Taken together the latter evidence with our present finding of no significant difference in the lag in intrinsic activity, an indirect estimation of the direction of the connection, between the pair of less interconnected networks (i.e. IC20-IC15), it is possible that the thalamic relay contributes the most to the cortical networks activity via the thalamocortical loops. Therefore, we hypothesize that a deficient thalamic activity in migraine between attacks, as highlighted by an increased FA [9], activates less the visuo-spatial system and medial visual cortical areas, which in turn leads to less activation of the DMN network (Fig. 4).

Clinical and experimental data indicate that the thalamus is a key structure in migraine pathophysiology. The thalamus was found to be implicated in many clinical [58–61] and neurophysiological features of migraine [62–64]. From animal experiments, it is known that the vast system of extrastriate and suprasylvian areas comprising the brain’s most important networks receive extensive projections from the lateral posterior-pulvinar thalamic complex, including the intralaminar nuclei [65, 66], that were recently reported to be reduced in volume in migraine patients scanned when attack-free [10]. It is important to take into account that these nuclei receive the most significant overlap of different sensory modalities [67]. This is of particular interest for migraine since the majority of evoked potential studies between attacks have shown abnormalities, such as deficit of habituation, for most sensory modalities: non-painful and painful somatosensory, auditory and visual [1]. The only notable exception is olfaction, the only sensory modality not relayed in the thalamus, for which brain and behavioural responses habituate normally in migraineurs [5].

As with all studies, our findings need to be considered with our study limitations and strengths. The small number of patients could make our study underpowered to reveal more subtle findings, such as correlation between clinical features and functional connectivity, although our cohort was sufficient to disclose strong statistical significance. A strength of the present study is our approach to study the dependencies between pairs of functional networks, since it allowed us to examine weak, but significant, connectivity among strongly connected networks.

## Conclusions

Overall, these results of RS-fMRI are in line with the concept of a global dysfunction in multisensory information processing and integration in migraine. The multimodal MRI data provide specifically structural and functional evidences for the involvement of the thalamus in the abnormal functional connectivity between different brain networks between attacks. Future work should attempt to clarify the role of the different networks with regard to migraine-associated multisensory phenomena, such as photophobia or allodynia, especially during the attack and in chronic migraine. It would also be of particular interest to verify whether the thalamocortical network dysfunctions are primary phenomena or secondary to a functional disconnection of the thalamus from the brainstem.

## Abbreviations

3 T: 3 Tesla
DMN: Default mode network
DTI: Diffusion tensor imaging
EEG: Electroencephalography
FA: Fractional anisotropy
HV: Healthy volunteer
IC: Independent component
ICA: Independent component analysis
MD: Mean diffusivity
MO: Migraine without aura
MRI: Magnetic resonance imaging
ROI: Region Of Interest
RS-fMRI: Resting state functional magnetic resonance imaging.
